# ADHD in DSM-5: a field trial in a large, representative sample of 18- to 19-year-old adults

**DOI:** 10.1017/S0033291714001470

**Published:** 2014-06-23

**Authors:** B. Matte, L. Anselmi, G. A. Salum, C. Kieling, H. Gonçalves, A. Menezes, E. H. Grevet, L. A. Rohde

**Affiliations:** 1 ADHD Out-patient Program, Hospital de Clinicas de Porto Alegre, Department of Psychiatry, Federal University of Rio Grande do Sul, Brazil; 2 Post-Graduate Program in Epidemiology, Federal University of Pelotas, Brazil; 3 National Institute of Developmental Psychiatry for Children and Adolescents, São Paulo, Brazil

**Keywords:** ADHD, diagnostic performance, DSM-5, epidemiology, prevalence

## Abstract

**Background:**

The DSM criteria for adult attention-deficit/hyperactivity disorder (ADHD) have not been tested in American Psychiatric Association (APA) field trials for either DSM-IV or DSM-5. This study aimed to assess: (*a*) the prevalence of ADHD according to DSM-5 criteria; (*b*) the factor solution that provides the best fit for ADHD symptoms; (*c*) the symptoms with the highest predictive value for clinical impairment; and (*d*) the best symptomatic threshold for each ADHD dimension (inattention and hyperactivity/impulsivity).

**Method:**

Trained psychologists evaluated 4000 young adults from the 1993 Pelotas Birth Cohort Study with an instrument covering all DSM-5 ADHD criteria. A series of confirmatory factor analyses (CFAs) tested the best factor structure. Complex logistic regressions assessed differential contributions of each symptom to clinical impairment. Receiver-operating characteristic (ROC) analyses tested which would be the best symptomatic cut-off in the number of symptoms for predicting impairment.

**Results:**

The prevalence of DSM-5 ADHD was 3.55% [95% confidence interval (CI) 2.98–4.12]. The estimated prevalence of DSM-IV ADHD was 2.8%. CFA revealed that a bifactor model with a single general factor and two specific factors provided the best fit for DSM-5 symptoms. Inattentive symptoms continued to be the most important predictors of impairment in adults. The best cut-offs were five symptoms of inattention and four symptoms of hyperactivity/impulsivity.

**Conclusions:**

Our results, combined with previous findings, suggest a 27% increase in the expected prevalence of ADHD among young adults, comparing DSM-IV to DSM-5 criteria. The DSM-5 symptomatic organization derived a similar factor structure for adults as DSM-IV symptoms. Data using DSM-5 criteria support lowering the symptomatic threshold for diagnosing ADHD in adults.

## Introduction

Attention-deficit/hyperactivity disorder (ADHD) is a neurodevelopmental disorder characterized by pervasive and persistent symptoms of inattention, hyperactivity and impulsivity, affecting individuals across the life cycle (Matte *et al.* 2012). Research and clinical data have documented that a significant proportion of children affected by the disorder continues to present ADHD symptoms and associated impairment during adulthood (Faraone *et al.* 2006; Barkley, 2010; Das *et al.* 2012). The two most comprehensive estimates of ADHD prevalence in adults suggest prevalence rates between 2.5% and 3.4% (Fayyad *et al.* 2007; Simon *et al.* 2009).

The diagnostic criteria for ADHD were not tested in adults in the field trials for DSM-IV (Matte *et al.* 2012), and ADHD was not among the disorders assessed in DSM-5 field trials in adults (Batstra & Frances, 2012). The DSM-5 criteria for ADHD kept the bidimensional structure of ADHD and the same list of symptoms, but proposed modifications that might impact on the ADHD diagnostic definition for adults, including: (*a*) a reduction in the number of required symptoms (cut-off point) in both ADHD dimensions (inattention and hyperactivity/impulsivity) from six to five in adults; (*b*) raising the age at onset from 7 to 12 years, and removing the requirement of impairment from this criterion; and (*c*) the addition of new clinical examples for symptoms. DSM-5 also proposed modifications that are relevant for all age ranges, such as defining pervasiveness as the presence of several ADHD symptoms (instead of impairment) in two or more settings, and the removal of autism/pervasive developmental disorder (PDD) as a specific exclusion (for a more comprehensive discussion on the rationale for these modifications, see Matte *et al.* 2012). These proposed changes in ADHD diagnostic criteria raised major concerns about potentially negative consequences, such as inflating prevalence rates of the disorder, overdiagnosis and overtreatment (Regier *et al.* 2013).

There are no epidemiological studies with non-referred subjects testing DSM-5 proposed changes. These studies are crucial for a proper evaluation of the prevalence rates and the natural distribution of the ADHD phenotype in the general population (Goodman *et al.* 1997). Additionally, information about how the specific diagnostic criteria impact on ADHD prevalence would be clinically useful, considering that ADHD is best conceptualized as a dimensional disorder (Willcutt *et al.* 2012). The performance of individual symptoms is even more relevant, considering that clinicians often match the patient's symptoms against a prototype based on some but not all diagnostic criteria, rather than using full DSM operational diagnostic criteria for making diagnoses (Maj, 2011; Jablensky, 2012).

Thus, our main objective was to assess the prevalence of ADHD according to DSM-5 criteria in a large, non-referred, representative population of young adults. We also addressed the individual impact of new criteria (such as the extension of the age of onset to 12 years and lower symptom cut-offs) on ADHD prevalence rates. In addition, because DSM-5 criteria displayed some rewording and new clinical examples for ADHD symptoms in adults, we also tested: (*a*) the factorial solution that provides the best fit for the DSM-5 ADHD symptoms, (*b*) the association of DSM-5 ADHD symptoms with clinical impairment, and (*c*) the best number (cut-off point) of DSM-5 ADHD symptoms to identify impaired adults in both ADHD dimensions.

## Method

### Participants

Participants in this study were young adults followed in the 1993 Pelotas Birth Cohort Study. Pelotas is a southern Brazilian city with around 327 000 inhabitants. A full description of the methodology is presented elsewhere (Victora *et al.* 2008). In brief, all 5249 live births in the city in 1993 whose mothers agreed to participate in the longitudinal study were considered eligible. The data used in this study were collected in 2011–2012, when the subjects were 18–19 years old. This follow-up assessment comprised all subjects who were located among the initial participants. The Institutional Review Board of the Federal University of Pelotas approved the study. Written informed consent was obtained from all subjects.

### Measures

Trained psychologists performed the entire diagnostic evaluation with the subjects. A general psychiatric assessment was performed with the Mini International Neuropsychiatric Interview (MINI), a short semi-structured diagnostic interview for DSM-IV and ICD-10 psychiatric disorders that provided prevalence estimates of common mental disorders. Because of logistic issues (i.e. the psychiatric interview was part of a larger follow-up assessment), only some MINI sections were performed. The most prevalent mood (major depression and bipolar) and anxiety (agoraphobia, social phobia, general anxiety) disorders were assessed. The MINI has a previously validated Portuguese version (Amorim, 2003). In primary health care in Brazil, the MINI exhibited *κ* values of 0.65–0.85, a sensitivity of 0.75–0.92 and a specificity of 0.90–0.99 when using the SCID applied by a psychiatrist as a parameter (Marques & Zuardi, 2008). Sociodemographic variables were collected at the same interview.

The ADHD assessment was performed with a structured interview that included 18 questions about DSM-5 ADHD symptoms (one question for each symptom). These questions were formulated exactly as proposed by the DSM-5 ADHD working group and were freely available during 2011 and 2012 at www.dsm5.org. Thus, we present here the performance of the current DSM-5 diagnostic criteria for ADHD (APA, 2013). DSM-5 defines ADHD in adults as the presence of at least five of nine symptoms of inattention and/or five of nine symptoms of hyperactivity/impulsivity. ADHD symptoms must cause clinical impairment, several of them must be present in more than one setting, and their age at onset should be before age 12 years.

The DSM-5 ADHD symptoms were rated as present or absent. Considering that this was a large population study, we initially applied a screening questionnaire using the same structure as the World Health Organization Adult ADHD Self-Report Scale (ASRS) screener for all subjects. The ASRS includes six questions about ADHD symptoms (four inattention items: ‘Does not follow through’, ‘Difficulty organizing tasks’, ‘Forgetful’, ‘Reluctant to engage in “mental” tasks’; and two hyperactivity items: ‘Fidgets’ and ‘On the go’). In a previous population study, the ASRS had 68.7% sensitivity, 99.5% specificity and 97.9% total classification accuracy, considering blind clinical assessment as the gold standard and using a cut-off of 4/6 screening symptoms (Kessler *et al.* 2005). In our questionnaire, those six ASRS questions were adapted to the exact DSM-5 wording. To enhance sensitivity, any subject with two or more positive questions among the six was considered screening positive, and answered 12 additional questions about the 12 remaining ADHD symptoms, along with other questions about additional criteria (symptom pervasiveness, age at onset before 12 years old, and clinical impairment). To assess symptom pervasiveness, subjects were asked if they presented symptoms in at least two of the three main settings: home, social and work/school environments. To assess the presence of ADHD symptoms before age 12, we asked ‘Did you have several of these symptoms before age 12?’ Clinical impairment specifically related to ADHD was measured using a question answered by the subjects at the end of the ADHD section of the assessment interview. The question asked was: ‘How much impairment your ADHD symptoms cause to your life?’ The options for answer were: none (0), mild impairment (1), moderate impairment (2), or severe impairment (3). For the current analyses, the presence of clinical impairment was operationally defined as having a score of 2 (moderate) or 3 (severe).

This strategy for assessing impairment was chosen to mimic approaches in clinical settings, in which clinicians tend to rely on the subject's general perception of impairment instead of on extensive evaluations of correlates of functioning. Thus, clinicians tend to assess ADHD impairment based on patient's perceptions (Rösler *et al.* 2006). Although the threshold for defining impairment is subjective and individual, clinicians diagnose ADHD even in cases without substantial impairment. Thus, the threshold of self-report moderate impairment tends to be conservative.

### Data analytic strategy: description of the sample

Sociodemographic and co-morbidity patterns were assessed both for DSM-5 ADHD cases and for subjects without ADHD. These two groups were compared using appropriate univariate tests.

### Assessment of ADHD prevalence and exploration of the impact of individual diagnostic criteria on ADHD prevalence rates

The consequences of using different symptom thresholds on ADHD prevalence rates were explored by calculating the proportion of subjects that would be defined as ADHD cases according to several potential symptomatic cut-offs. We also applied the ADHD additional criteria consecutively to examine their individual impact on ADHD prevalence rates. For these prevalence assessments, we used data from the entire sample; the subjects who screened negative for ADHD (less than two out of six positive screening questions) were assumed to be negative for ADHD. Moreover, we estimated a comparison between DSM-IV and DSM-5 ADHD prevalence in our sample. This was an indirect comparison because we could not assess either ADHD symptoms with DSM-IV wording or the presence of impairment of symptoms before 7 years (as DSM-IV required). To derive an ADHD diagnosis according to DSM-IV criteria, we considered data from this sample using the 6/9 cut-point proposed by DSM-IV. To deal with age-of-onset criterion modification while avoiding the effect of recall bias in a cross-sectional study asking for the exact date of beginning ADHD symptoms, we relied on a previous study documenting an increase of 0.1% in ADHD prevalence rates with this DSM criterion B modification. It is important to note that this rate was obtained in a longitudinal assessment (Polanczyk *et al.* 2010). Thus, we estimated the DSM-IV prevalence in this sample as the ADHD prevalence for the 6/9 cut-point with an additional 0.1% discount.

### Assessment of the factor solution that provides the best fit for ADHD symptoms

The factor structure of the 18 DSM-5 ADHD symptoms was tested with confirmatory factor analysis (CFA) in the subsample that screened positive for ADHD (at least 2/6 positive screening questions) and completed the entire ADHD assessment. Several CFA models were fitted to the data: (*a*) a one-factor model; (*b*) a correlated two-factor model; (*c*) a correlated three-factor model; (*d*) a second-order two-factor model; (*e*) a second-order three-factor model; (*f*) a bifactor model with one general and two specific factors; (*g*) a bifactor model with one general and three specific factors; and (*h*) an incomplete bifactor model with one general and two specific factors (in this model the hyperactivity factor is suppressed as all hyperactivity symptoms are explained by the general ADHD factor). This strategy allowed us to determine which factor solution provided the best fit for DSM-5 ADHD symptoms. CFA models were fitted to polychoric correlations among the symptoms using the weighted least squares means and variance adjusted (WLSMV) estimator implemented in Mplus 7.0 (Muthén & Muthén, 2012). The goodness of fit was assessed using the following fit indices: *χ*^2^, the weighted root mean square residual (WRMR), the Comparative Fit Index (CFI), the Tucker–Lewis Index (TLI) and the root mean square error of approximation (RMSEA). To demonstrate good fit to the data, an estimated model should have a WRMR ⩽0.9 (Muthén & Muthén, 2012), RMSEA ⩽0.06 and CFI and TLI ⩾0.95 (Hu & Bentler, 1999). We compared nested models directly using the robust chi-square difference test with mean and variance adjusted test statistics (Asparouhov & Muthén, 2006). We also provided the differences between models with regard to Akaike information criterion (ΔAIC) and Bayesian information criterion (ΔBIC), using the robust maximum likelihood estimator (MLE). Lower values for AIC and BIC indicate a better fit. Values of ΔAIC and ΔBIC ⩽10 indicate overwhelming support for the models with lower values (Burnham, 2004). To reduce the number of statistical comparisons, we first identified the best models within the correlated models (including the unidimensional model), the bifactor models and the second-order models. We then compared the best models from each kind of factor structure.

### Assessment of the association of ADHD symptoms with clinical impairment

A three-step approach was implemented to examine which individual DSM-5 ADHD symptoms are the best predictors of moderate to severe clinical impairment. These analyses were also performed only in the subsample that screened positive for ADHD. First, the bivariate association between symptoms and clinical impairment was assessed with the *χ*^2^ test and unadjusted odds ratio (OR) estimates, which allowed ranking the DSM-5 symptoms according to their OR related to clinical impairment. Second, a binary stepwise logistic regression model was performed, considering clinical impairment as the dependent variable and all DSM-5 ADHD symptoms as independent variables. This analysis depicted how many, and which, DSM-5 symptoms were independently associated with clinical impairment after controlling for the other DSM-5 symptoms. Third, all-possible-subsets (APS) logistic regression analysis was used to confirm the set of DSM-5 symptoms that best predicted clinical impairment. The APS analysis helped to select the best subset from a larger set of predictors. In such situations, different subsets might have almost equivalent associations with the outcome, and conventional stepwise regression analysis might select a suboptimal subset because of minor differences in bivariate associations. The APS analysis avoids this problem as it generates results for a large number of different models with a fixed number of predictors, which were determined by the earlier stepwise logistic regression analysis. The APS analysis also ranks the best subsets according to their association with the outcome (using *χ*^2^ values as ranking criteria). Once the ranking of subsets is known, the researcher can select the predictors that are more consistent across the top-ranked subsets. In our APS analyses, we followed the procedures of Kessler *et al.* (2010) but used clinical impairment as the dependent variable instead of ADHD diagnosis.

### Assessment of the best number of ADHD symptoms (cut-off point) to identify clinical impairment

Receiver-operating characteristic (ROC) analyses tested which would be the best cut-off (i.e. the best balance between sensitivity and specificity assuming an equal chance of false-positive and false-negative errors in selecting the optimal cut-point) for the number of DSM-5 ADHD symptoms to predict clinical impairment, for both inattention and hyperactivity/impulsivity dimensions. For these analyses, we initially assumed that those with no positive symptom among the six screening symptoms would be negative for the remaining 12 symptoms. To test this assumption, we simulated the performance of the screening questions in a real dataset that had all the 18 symptoms without any skipping rule for a sample of children from the same Brazilian region (*n* = 1255; 6–12-year-olds) (Salum *et al.* unpublished observations). For children with no positive symptoms in the questions that were part of the screening instrument, the vast majority (86.9%) had no additional symptoms. Thus, we included subjects with no positive symptoms in the screening instrument in our first set of ROC analyses. However, we did not include subjects with one positive symptom among the six screening items in this first set of analyses because predictions of the number of other positive symptoms and impairment from the symptoms would be impossible. Next, we performed the same analyses only in the ADHD screening positive subsample.

For all analyses, a 5% significance level for two-tailed test was adopted, unless stated otherwise. The analyses were performed in SAS version 9.0 (APS analysis), Signal Detection Software ROC4 (ROC analyses), Mplus version 7.0 (CFA) and IBM SPSS version 20 (other analyses).

## Results

### Description of the sample

All subjects were in the age range between 18 and 19 years at the time of the interview. A total of 4106 cohort members were located (81.4% of the initial sample), and 4000 subjects provided information on ADHD, co-morbidities and sociodemographics. Of these, 1329 subjects (33.2% of this study sample) screened positive for ADHD and provided information about all ADHD criteria. Sociodemographic and co-morbidity profiles for ADHD cases and subjects without ADHD are shown in Table 1.

**Table 1.** Sociodemographics and co-morbidity profile of DSM-5 ADHD cases and subjects without ADHD (n=4000)

	Subjects without ADHD	ADHD cases^a^
*n* (%)	3858	142 (3.55)
Gender (%)		
Male	49.10^b^	39.40^b^
Self-reported skin color (%)		
White	64.10	59.30
Black	14.50	16.30
Brown	17.60	23.00
Other (Indigenous Brazilian/Asian)	3.70^b^	1.50^b^
Marital status (%)		
Living with partner	10.90	14.30
Academic achievement		
Years of schooling (mean±s.d.)	8.63±2.29	7.96±2.30
ADHD presentation (%)		
Inattentive	n.a.	46.50
Hyperactive/impulsive	n.a.	12.70
Combined	n.a.	40.80
Impairment related to ADHD symptoms (%)		
Moderate impairment	n.a.	60.60
Severe impairment	n.a.	39.40
Co-morbidities (%)		
Major depressive disorder	3.97	6.34
Bipolar disorder	1.74	2.82
Anxiety disorders	39.70	37.6

ADHD, Attention-deficit/hyperactivity disorder; n.a., not applicable; s.d., standard deviation.

a ADHD cases are subjects with at least five of nine inattention and/or five of nine hyperactivity symptoms + symptom onset before age 12 + symptoms in more than one setting + moderate or severe impairment related to ADHD symptoms.

b Statistical difference between ADHD and non-ADHD groups (*p* < 0.05). For the 66 ADHD subjects with inattentive presentation, mean ± s.d. of number of hyperactivity/impulsivity symptoms was 2.65 ± 1.2. For the 18 ADHD subjects with hyperactive/impulsive presentation, mean ± s.d. of number of inattention symptoms was 3.22 ± 0.9.

### Assessment of ADHD prevalence and exploration of the impact of individual diagnostic criteria on ADHD prevalence rates

According to full DSM-5 criteria, the prevalence of ADHD was 3.55% [95% confidence interval (CI) 2.98–4.12]. The prevalence of inattentive, hyperactive/impulsive and combined presentations was 1.65, 0.45 and 1.45% respectively. The effects on ADHD prevalence rates of using different symptom cut-off points for assigning a diagnosis are displayed in Table 2. This table also shows the impact on prevalence rates of sequentially applying the ADHD additional criteria. In general, the most significant decrease in ADHD prevalence was determined by age-at-onset criterion, which had a larger effect on prevalence rates than the symptom cut-off or the impairment criteria. Note that ADHD prevalence rates had overlapping CIs with four, five or six symptom cut-offs (Table 2). If severe (rather than at least moderate) clinical impairment was used as an ADHD additional criterion, the prevalence of ADHD would be 1.4% (95% CI 1.04–1.76%). The ADHD prevalence rate with at least six symptoms in any dimension was estimated to be 2.9% (95% CI 2.38–3.42%) in our sample (Table 2). Discounting the impact of the modification of age-at-onset criterion estimated at 0.1%, we would end up with a DSM-IV ADHD prevalence rate of about 2.8%. We also ran secondary analyses comparing the three groups (subjects without ADHD, ADHD cases based on both DSM-IV and DSM-5, and new ADHD cases based only on DSM-5 criteria) with regard to male/female ratio, impairment and co-morbidity. There are no differences between the two groups of cases on impairment (*p* = 0.73) and both groups differed from the subjects without ADHD (*p* < 0.001). Both groups of cases also presented similar male/female ratios (39.3% *versus* 40%) and rates of co-morbidity with anxiety disorders (37.1% and 40%). However, new cases identified only by DSM-5 had a higher prevalence of co-morbid mood disorders (16% and 7.7%).

**Table 2.** Prevalence (95% CI) of ADHD across several symptom cut-offs and sequential application of additional ADHD criteria (n = 4000)

	Symptom cut-off only (without additional criteria)	Symptom cut-off + symptoms in two or more settings (A)	Symptom cut-off + age of onset < 12 years (B)	Symptom cut-off + at least moderate impairment (score ⩾ 2 on a scale 0–3)^a^	Symptom cut-off + A and B + at least moderate impairment
At least 2/9 inattention symptoms or 2/9 hyperactivity/impulsivity symptoms	33.2 (31.74–34.66)	25.2 (23.85–26.55)	6.7 (5.93–7.47)	18.7 (17.49–19.91)	3.9 (3.3–4.5)
At least 3/9 inattention symptoms or 3/9 hyperactivity/impulsivity symptoms	32.3 (30.85–33.75)	24.5 (23.17–25.83)	6.5 (5.74–7.26)	18.4 (17.2–19.6)	3.9 (3.3–4.5)
At least 4/9 inattention symptoms or 4/9 hyperactivity/impulsivity symptoms	29.4 (27.99–30.81)	22.8 (21.5–24.1)	6.3 (5.55–7.05)	17.6 (16.42–18.78)	3.8 (3.21–4.39)
At least 5/9 inattention symptoms or 5/9 hyperactivity/impulsivity symptoms	23.7 (22.38–25.02)	18.6 (17.39–19.81)	5.4 (4.7–6.1)	15.1 (13.99–16.21)	3.55 (2.98–4.12)
At least 6/9 inattention symptoms or 6/9 hyperactivity/impulsivity symptoms	17.6 (16.42–18.78)	14.1 (13.02–15.18)	4.2 (3.58–4.82)	12 (10.99–13.01)	2.9 (2.38–3.42)
At least 7/9 inattention symptoms or 7/9 hyperactivity/impulsivity symptoms	10.9 (9.93–11.87)	9 (8.11–9.89)	2.6 (2.11–3.09)	7.9 (7.06–8.74)	1.9 (1.48–2.32)

ADHD, Attention-deficit/hyperactivity disorder; CI, confidence interval.

a Impairment related to the ADHD symptoms.

Additional data for subjects presenting with five of nine inattentive + five of nine hyperactive/impulsive symptoms are available upon request.

### Assessment of the factor solution that provides the best fit for ADHD symptoms

The data fit indexes for several factorial models tested in CFA are presented in Table 3. The model with the best factor structure for DSM-5 symptoms was the bifactor model with one general factor and two specific factors (RMSEA 0.045, 90% CI 0.040–0.049; CFI 0.875; TLI 0.837; WRMR 1.492). The most important implication from these findings is that a general factor substantially influences all of the ADHD symptoms whereas the specific factors account for additional variance related to inattention and hyperactivity/impulsivity.

**Table 3.** Confirmatory factor analysis (CFA) of 18 DSM-5 ADHD symptoms with fit indexes for different models of ADHD and model comparison (n = 1329)

Individual models	FP	*χ*^2^	df	RMSEA (90% CI)	CFI	TLI	WRMR	AIC	BIC
One-factor (1F)	36	1312.284	135	0.081 (0.077–0.085)	0.530	0.467	2.751	28299.938	28486.856
Correlated two-factor (C_2F_)	37	843.561	134	0.063 (0.059–0.067)	0.717	0.676	2.225	27968.951	28161.061
Correlated three-factor (C_3F_)	39	820.780	132	0.063 (0.059–0.067)	0.725	0.681	2.180	27935.872	28138.367
**Bifactor (one general, two specific, B**_**1G2S**_**)**	**54**	**429.482**	**117**	**0.045 (0.04–0.049)**	**0.875**	**0.837**	**1.492**	**27655.448**	**27973.256**
Bifactor (one general, three specific)	No convergence								
Bifactor incomplete (B_inc_)	48	727.001	123	0.061 (0.057–0.065)	0.759	0.700	1.983	27864.720	28147.216
Second-order two-factor	No convergence								
Second-order three-factor (2^nd^_3F_)	39	820.780	132	0.063 (0.059–0.067)	0.725	0.681	2.180	27935.819	28138.314
Model comparison	Δ*χ*^2^	df	*p* value	ΔAIC	ΔBIC	
Within correlated models									
1F *v*. C_2F_			179.322	1	<0.0001	330.99	325.79	C_2F_ is better than 1F	
C_2F_ *v*. C_3F_			22.040	2	<0.0001	33.08	22.69	C_3F_ is better than C_2F_	
Within bifactor models									
B_1G2S_ *v*. B_inc_			220.024	6	<0.0001	−209.27	−173.96	B_1G2S_ is better than B_inc_	
Between models with best fit									
C_3F_ (best correlated) *v*. B_1G2S_ (best bifactor)			305.820	15	<0.0001	280.42	165.11	B_1G2S_ is better than C_3F_	
C_3F_ (best correlated) *v*. 2^nd^_3F_			–					–	
B_1G2S_ (best bifactor) *v*. 2^nd^_3F_			305.820	15	<0.0001	−280.37	−165.06	B_1G2S_ is better than 2^nd^_3F_	

ADHD, Attention-deficit/hyperactivity disorder; FP, free parameters; df, degrees of freedom; RMSEA, root mean square error of approximation; CI, confidence interval; CFI, Comparative Fit Index; TLI, Tucker–Lewis Index; WRMR, weighted root mean square residual; AIC, Akaike information criterion; BIC, Bayesian information criterion.

Parameters were estimated using mean and variance adjusted weighted least squares (WLSMV), except for AIC and BIC, which were estimated using the robust maximum likelihood estimator (MLE). Δ*χ*^2^ represents the robust chi-square difference test with mean and variance adjusted test statistics. ΔAIC and ΔBIC represent the difference between AIC and BIC for each model comparison: values ⩾10 indicate overwhelming support for the model with the lower AIC and BIC values. Bold values indicate the model that provided the best fit for the data.

### Assessment of the association of ADHD symptoms with clinical impairment

The results for the performance of the 18 DSM-5 symptoms in predicting moderate to severe clinical impairment are presented in Table 4. According to the unadjusted OR ranking, there were four inattention symptoms among the top-five predicting items. In the conventional logistic regression analysis, seven symptoms (five inattention and two hyperactivity items) remained as independent predictors of clinical impairment (adjusted *R*^2^ = 0.123). In the APS regression, five inattention (‘Difficulty sustaining attention’, ‘Difficulty organizing tasks’, ‘Easily distracted’, ‘Loses objects’, ‘Fails to give close attention to details’) and only two hyperactivity symptoms (‘On the go’, ‘Gets up’) were present in more than half of the top-10 subsets of seven symptoms. In all the analyses, inattention items performed better than hyperactivity/impulsivity items in predicting clinical impairment.

**Table 4.** Association of individual DSM-5 ADHD symptoms with clinical impairment (n = 1329)

DSM-5 symptoms	Endorsement (%)		Association with at least moderate clinical impairment^a^ (score ⩾2, scale 0–3)			
	Screening positive subjects (1329)	ADHD cases (142)	Unadjusted OR (95% CI)	Unadjusted OR ranking	Adjusted OR (95% CI)	APS
01. Fails to give close attention to details	67.2	75.4	1.73 (1.37–2.18)	6	1.36 (1.07–1.75)	**6**
02. Difficulty sustaining attention	64.9	83.1	2.06 (1.63–2.59)	*2*	1.52 (1.18–1.94)	**10**
03. Does not seem to listen	49.1	60.6	1.82 (1.46–2.27)	*5*	n.s.	3
04. Does not follow through	46	56.3	1.73 (1.39–2.16)	6	n.s.	4
05. Difficulty organizing tasks	49.8	64.8	1.83 (1.47–2.28)	*4*	1.66 (1.31–2.09)	**10**
06. Reluctant to engage in ‘mental’ tasks	75.3	81.7	1.30 (1.01–1.66)	14	n.s.	0
07. Loses objects	30.2	43	1.61 (1.26–2.04)	9	1.41 (1.09–1.82)	**8**
08. Easily distracted	81.7	90.1	2.51 (1.89–3.35)	*1*	2.13 (1.58–2.87)	**10**
09. Forgetful	61.1	68.3	1.37 (1.09–1.71)	11	n.s.	0
10. Fidgets	83.1	86.6	0.90 (0.67–1.20)	n.s.	n.s.	1
11. Gets up	34.4	52.8	1.88 (1.49–2.38)	*3*	1.43 (1.11–1.84)	**6**
12. Runs about	56.5	73.9	1.64 (1.32–2.04)	8	n.s.	2
13. Excessively loud	38.8	48.6	1.28 (1.02–1.60)	15	n.s.	0
14. On the go	31.6	53.5	1.57 (1.24–1.99)	10	1.47 (1.14–1.89)	**10**
15. Talks excessively	30.5	36.6	1.35 (1.07–1.72)	12	n.s.	0
16. Blurts out answers	31.6	43.7	1.28 (1.01–1.62)	15	n.s.	0
17. Difficulty waiting his turn	39.4	45.1	1.31 (1.06–1.64)	13	n.s.	0
18. Interrupts or intrudes	9.2	12.7	1.31 (0.89–1.92)	n.s.	n.s.	0

ADHD, Attention-deficit/hyperactivity disorder; OR, odds ratio; CI, confidence interval; APS, all possible subsets; n.s., non-significant association between ADHD symptom and clinical impairment.

These analyses were performed only for the 1329 subjects who screened positive for ADHD (at least two positive screening questions) and provided information on all 18 ADHD symptoms. Bold values indicate the 7 symptoms that were more consistent across the top-10 ranked subsets of *n*=7 symptoms in APS regression. According to this criterion, these highlighted symptoms would be the best predictors of impairment.

a Impairment related to the ADHD symptoms. Unadjusted ORs from the *χ*^2^ test. Adjusted ORs from conventional binary regression analysis. APS results display the number of times the symptoms appear among the top-10 ranked subsets of seven symptoms (according to general *χ*^2^).

### Assessment of the best number of ADHD symptoms (cut-off point) to identify clinical impairment

ROC analyses were performed with both inattention and hyperactivity/impulsivity symptoms, considering the presence of moderate to severe clinical impairment as the gold standard. In the model including subjects with no positive symptoms in the screening instrument, the best inattention cut-off for predicting clinical impairment was four symptoms [sensitivity 88% and specificity 70.9%; area under the curve (AUC) for the ROC analysis 0.860, 95% CI 0.845–0.875]. For hyperactivity/impulsivity, the best cut-off was one symptom (sensitivity 96% and specificity 61%; AUC 0.819, 95% CI 0.802–0.837). When only the ADHD screening positive subjects were included, the best inattention cut-off for predicting clinical impairment was five symptoms (sensitivity 73.4% and specificity 49.8%; AUC 0.658, 95% CI 0.629–0.688). For hyperactivity/impulsivity, the best cut-off was four symptoms (sensitivity 54.2% and specificity 61.4%; AUC 0.588, 95% CI 0.558–0.619). The sensitivity and specificity for other cut-off points are available upon request.

## Discussion

As far as we are aware, this is the first comprehensive evaluation of DSM-5 ADHD criteria in a large, representative, population-based sample of adult subjects. In general, our results suggest that the new DSM-5 ADHD criteria would determine a 27% increase in the prevalence rate of ADHD, compared with DSM-IV criteria (3.55% *v.* 2.8%). However, the new criteria did not impact substantially on the factor structure of the disorder. In addition, our data support previous DSM-IV findings suggesting that inattentive symptoms are core in the ADHD diagnostic definition in adults, and that there are potential benefits in lowering the symptomatic threshold for diagnosing ADHD in adults.

Our clinical and sociodemographic data concur with previous findings. The developmental decrease in hyperactive symptoms determines that ADHD inattentive is often the most frequent clinical presentation seen in clinical and epidemiological samples of adults (Grevet *et al.* 2006; Barkley, 2010). Although Kessler *et al*. (2006) have found a male/female ratio of around 1.6, previous studies in adults did not find consistent gender effects, as seen in children (Kooij *et al.* 2005). As in our study, a preponderance of females was detected when the DSM-IV symptomatic cut-off was reduced from 6/9 to 4/9 in a population study in The Netherlands (Kooij *et al.* 2005). Our co-morbidity rates with anxiety and mood disorder were lower than those detected by Kessler *et al.* (2005), probably reflecting the fact that we assessed young adults differently; the study by Kessler *et al.* assessed individuals up to 44 years of age. Although the lack of significant difference in academic achievement between cases and subjects without ADHD in our study might reflect the young age of the adults assessed, Kessler *et al*. (2005) also did not find a significant difference in education in the National Comorbidity Survey Replication (NCS-R). However, other studies have found a lower academic achievement for subjects with ADHD (e.g. Ebejer *et al.* 2012).

When all additional criteria (symptom pervasiveness, age at onset before age 12 years, and moderate/severe impairment) besides symptom threshold were used, the prevalence of ADHD ranged from 2.9% (with a cut-off of six symptoms in any dimension) to 3.8% (with a four-symptom cut-off). These prevalence rates are not very different from the rates found in previous DSM-IV-based population studies in the USA. A telephone survey of 966 adults (Faraone & Biederman, 2005) found a 2.9% prevalence of ADHD. A rate of ADHD of 4.4% was reported in a US nationally representative household survey with 3199 adults (Kessler *et al.* 2006). International estimates of ADHD prevalence in adults were also in the same range (Simon *et al.* 2009). In a comprehensive assessment in 10 different countries with 11 422 adults, a mathematical model of multiple imputations was used to derive a pooled ADHD prevalence of 3.4% (Fayyad *et al.* 2007). However, lower prevalence rates have been found in some studies (Kooij *et al.* 2005; Medina-Mora *et al.* 2005; Ebejer *et al.* 2012). These lower rates are closer to the rates that our study found when severe impairment was required as an additional criterion for ADHD (1.4%). Because of differences in methodology, comparisons among different prevalence studies are always difficult (see Polanczyk *et al.* 2007). The relevant issue here is that, by using the same methodology and in the same sample, we found an estimated increase of 27% in the prevalence rate of ADHD on going from DSM-IV to DSM-5. It is important to note that ADHD cases by the definition assumed in this study reported at least moderate impairment caused by the symptoms, independently of the diagnostic system used. In addition, ADHD cases defined only by DSM-5 did not present a significantly different impairment score than those identified by both DSM versions. Thus, DSM-5 criteria might be extending the diagnosis to people suffering with the disorder who were not assigned a place in the classification system according to the previous DSM-IV. Future studies need to address how to decide where to put the diagnostic threshold in dimensional disorders such as ADHD, where investigations have been documenting for a long time that the linear increase in symptoms seems to determine augmentation of impairment (Fergusson *et al.* 1997).

Our results also underscore the importance of addressing all ADHD criteria, not just symptom count. In particular, the impact of applying the DSM-5 age-at-onset criterion on reducing ADHD prevalence is relevant: considering only a five-symptom cut-off in any dimension, the ADHD endorsement rate would be 23.7% (95% CI 22.38–25.02). Adding the age-at-onset criterion alone (without other additional criteria) would decrease this rate to 5.4% (95% CI 4.7–6.1). As expected, a similar result emerged for the impairment criterion: adding the moderate impairment criterion alone would increase the endorsement rate to 15.1% (95% CI 13.99–16.21). These additional criteria were more relevant for making the prevalence closer to the one expected than any potential variations in symptom cut-off criterion. Taken together, these results might serve as clinical rationale for emphasizing the importance of carefully addressing ADHD additional criteria (especially age at onset and impairment) during the diagnostic evaluation.

CFAs with the DSM-5 symptoms found a bifactor model with one general and two specific factors as the best fit for the data. A bifactor model was also the best fit in many DSM-IV-based previous studies (Dumenci *et al.* 2004; Toplak *et al.* 2009, 2012; Martel *et al.* 2010, 2011; Gibbins *et al.* 2012; Normand *et al.* 2012; Ullebø *et al.* 2012), suggesting that the proposed rewording and new clinical examples would not change the expected factorial structure of ADHD symptoms. This model is in line with multiple pathway conceptions of the disorder (Nigg *et al.* 2005) and better explains symptomatic overlap/distinctiveness and stability/change, accounting more clearly for disorder heterogeneity.

Regarding the performance of DSM-5 symptoms in predicting ADHD-related impairment, the three-step approach yielded consistent results, suggesting that inattention symptoms, as a group, are more closely associated with impairment than hyperactivity/impulsivity symptoms. This pattern does not seem to be influenced by a higher endorsement rate for inattention symptoms. If we rank the 18 ADHD symptoms according to their frequency in ADHD patients, the seven symptoms significantly associated with impairment are ranked as follows: 1st, 3rd, 5th, 8th, 11th, 12th and 16th. Thus, it does not seem that those significantly associated with impairment were only distributed in the first ranks of frequency. To analyze this issue statistically, we assessed the correlation of the 18 symptom ranks in terms of frequency with their ranks in terms of the number of times they appeared as part of the top-10 subsets of seven symptoms predicting impairment in APS analyses. No significant association was found (*p* > 0.10). These results stress the relevance of inattention as a predictor of clinical impairment in adults. Although the specific list varies across studies, inattentive symptoms as a group are usually reported as the best predictors of ADHD-related impairment in previous investigations. Barkley *et al.* (2008) reported that the five ADHD symptoms with the strongest associations with the presence of any impairment were inattentive symptoms. A population-based study by Das *et al.* (2012) also documented that inattentive symptoms were the most strongly associated with clinical impairment.

Our ROC analyses, independently of the strategy used, found that a lower symptom threshold than the one proposed by DSM-IV determined the best balance between sensitivity and specificity for predicting at least moderate impairment for both inattention and hyperactivity/impulsivity dimensions. These findings concur with previous DSM-IV-based studies suggesting that a lower threshold of symptoms might be adequate in adult ADHD samples (Murphy & Barkley, 1996; Solanto *et al.* 2012). Kooij *et al.* (2005) reported that subjects with four or more DSM-IV symptoms in any ADHD dimension were significantly more impaired than subjects with fewer symptoms in a large, population-based sample in The Netherlands. Moreover, an investigation by Hoogman *et al.* (2012) suggested similar brain volumetric measures in adults presenting with a lower symptom threshold of four symptoms and subjects with six or more symptoms in any ADHD dimension. Both groups differed from those with fewer ADHD inattentive or hyperactive/impulsive symptoms.

Our study has some strengths and also methodological limitations related to logistic issues. To the best of our knowledge, this is the first population study assessing DSM-5 ADHD criteria. We assessed young adults, and all previous DSM-IV and DSM-5 field trials in adults have not addressed ADHD. Our data were derived from a large, representative, population-based sample. Finally, we implemented assessment methods that could mimic clinical assessment in the real world, as far as possible in an epidemiological investigation. Regarding limitations, our subjects were all 18–19 years old and our data might not be fully generalized to older adults. Our diagnostic process was performed only with the subjects and did not aggregate information from significant others, a strategy that could have minimized the risk of under-reporting ADHD symptoms, and might have improved the reliability of our retrospective assessment of ADHD symptoms before age 12. Our impairment measure was based only on the subject's perspective; a rater-derived score based on functional correlates was not used. It is important to bear in mind that previous investigations have found high agreement between self and parent information for ADHD diagnosis in adults (see Murphy & Schachar, 2000), and clinicians tend to see young adult patients without parents and to rely on self-perception about impairment more than on scales. Thus, we are confident that our strategy of assessment adequately mimics the scenario seen in a clinical setting. However, our data are probably more applicable to scenarios where diagnosis relies on self-report. We could not assess some important ADHD co-morbidities, such as other externalizing disorders, anti-social personality disorder or substance-use disorder. Moreover, we used a screening tool for ADHD, and 2671 subjects (66.8% of the total sample) presented a negative screening for the disorder and therefore answered only the six screening questions. This high proportion of screening-negative subjects posed some difficulties to our data analyses. Those analyses that required information about individual symptoms (CFA, association between individual symptoms and impairment) had to be performed only with the subsample that screened positive and provided complete information about all ADHD symptoms (*n* = 1329). For our prevalence assessment, we assumed that those who screened negative for ADHD did not have the disorder. This assumption allowed us to assess the prevalence of ADHD for the entire population sample, but it is theoretically possible (although clinically unlikely) that our approach missed some ADHD cases. To deal with this possibility, we performed a mathematical modeling for imputing data for the 1833 subjects who had only one screening positive symptom based on the patterns of response of those that had two screening positive symptoms and therefore provided information on all 18 symptoms (data available upon request). The prevalence rate found was slightly higher, as expected (3.9%, 95% CI 3.3–4.5). Finally, the AUC for our ROC analyses when only the ADHD screening positive subsample was included showed a diagnostic performance substantially lower than that detected when the entire sample was considered (i.e. individuals in the lower extreme of the symptomatic presentation are not excluded). In other words, these analyses are more likely to reflect the clinical reality of differentiating clinical cases from those with some level of symptoms including subthreshold cases.

Overall, our findings suggest that the DSM-5 changes in ADHD criteria determine a 27% increase in ADHD prevalence rates in adults, even when all diagnostic criteria (particularly age-at-onset and impairment criteria) are included in the evaluation process. The results reinforce the notion that using new wording and clinical examples would not change either the expected factorial structure of ADHD symptoms or the stronger association between inattention and clinical impairment found in adults. Moreover, our study reinforces the view that a lower cut-off point for the number of inattentive and/or hyperactive/impulsive symptoms is more suitable for adult patients.
